# Machine learning modelling of sonochemical systems using physically-derived dimensionless groups

**DOI:** 10.1016/j.ultsonch.2025.107593

**Published:** 2025-09-30

**Authors:** Yucheng Zhu, Ruosi Zhang, Xueliang Zhu, Xuhai Pan, Michael Short, Lian X. Liu, Madeleine J. Bussemaker

**Affiliations:** a School of Chemistry and Chemical Engineering, University of Surrey, Guildford, United Kingdom; b College of Safety Science and Engineering, Nanjing Tech University, Nanjing, China

**Keywords:** Sonochemistry, Machine learning, Dimensionless modelling, CatBoost, Mechanism visualisation

## Abstract

Sonochemistry involves complex multiparametric effects and nonlinear interactions that challenge conventional analysis and modelling approaches, especially when extrapolating across systems. Current models mainly depend on dimensional input variables, limiting generalisability and interpretability. This work proposes a machine learning strategy that integrates physically derived dimensionless variables (*Π*-terms) into a categorical boosting (CatBoost) algorithm to overcome these limitations. Four representative sonochemical outputs, namely sonochemiluminescence (SCL) intensity, SCL area, and ultrasonic oxidation from iodide oxidation radicals (IORS) and both IORS and H_2_O_2_, were selected as model targets. Seven supervised learning algorithms, including k-nearest neighbours (KNN), linear regression, support vector regression (SVR), random forest, gradient boosting, extreme gradient boosting (XGBoost), and CatBoost, were evaluated, with tree-based models exhibiting superior performance. CatBoost was finally selected as the baseline model. Regression models using the same *Π*-terms achieved R^2^ = 0.67–0.90 on the full dataset but required dataset-specific corrections to predict independent validation sets. However, the machine learning framework reached higher predictive accuracy (R^2^ = 0.87––0.95 on the reserved test set) and generalised to external validation datasets without additional corrections. Furthermore, a direct comparison between dimensional and dimensionless input strategies showed that dimensionless-input models provided superior generalisability and task-to-task consistency, alleviating plateau effects observed in dimensional models and yielding more stable feature attributions. SHAP analysis highlighted variables associated with cavitation thermal buffering and energy input scaling (>50 % combined importance across tasks), offering mechanistic insights into these nonlinear behaviours that regression could not capture.

## 1 Introduction

Sonochemistry is used to describe chemical transformations that arise from ultrasonic cavitation without needing to introduce extra chemicals [1]. Due to its efficiency and environmental sustainability, sonochemistry has potential in various applications, including ultrasonic-assisted advanced oxidation processes [2], environmental remediation [3], material synthesis [4], and biomedical engineering [5]. Sonochemical activity is governed by a complex interplay of multiple parameters. These factors are commonly categorized into primary parameters (e.g., pressure amplitude, frequency, and reactor design), which define the fundamental cavitation behaviour, and secondary parameters (e.g., gas composition, liquid properties and additives), which modulate the chemical environment [6]. The dynamic behaviour of cavitation bubbles, including bubble generation and the distribution of collapse energy, is directly or indirectly influenced by these parameters, thereby affecting the overall reaction performance of the acoustic system [7,8]. However, optimising one parameter in isolation provides limited guidance for real-world systems, where multiple variables interact nonlinearly to determine the final sonochemical outcomes [7,9]. It is essential to seek approaches capable of capturing the multivariate and nonlinear characteristics of sonochemical responses to gain deeper mechanistic insights and enable robust system-level optimisation in such a chaotic system.

Conventional modelling approaches rely on predefined mathematical assumptions like linear relationships or steady-state conditions [10]. These approaches also require manual construction of the underlying structure based on prior knowledge of system dynamics and variable interactions. However, such methods generally struggle to capture the full complexity of sonochemical systems, which are inherently nonlinear and involve multiple interacting variables [9]. In recent years, machine learning has developed as a strong option, effectively understanding complex patterns from high-dimensional data without presupposing existing functional forms [11]. Machine learning methods such as random forests, extreme gradient boosting (XGBoost), and gradient boosting algorithms have demonstrated strong predictive performance in sonochemistry-related tasks, including bubble collapse dynamics [9], degradation kinetics of micropollutants [12], and process optimisation in ultrasound-assisted advanced oxidation processes [13]. Moreover, several studies have incorporated explainable machine learning tools, such as SHAP (Shapley Additive exPlanations), to attribute predictions to input features, enabling insights into the factors governing system performance [14,15]. However, such tools operate at the feature attribution level and do not inherently uncover the underlying physical mechanisms. Despite their strong predictive capabilities, most machine learning-based sonochemical models primarily rely on dimensional input variables (e.g., frequency, power density, molecular weight), overlooking the physical structure and dimensional consistency of input information. Dimensional consistency involves constructing input features that preserve physical units and scaling relationships, ensuring that models remain valid and interpretable across varying experimental or operational contexts. Pure machine learning approaches suffer from limited interpretability and offer little insight into the underlying physical mechanisms, functioning as black boxes [16]. This disconnect between predictive performance and physical understanding limits generalisability across systems [17] and highlights the need for physics-guided modelling.

Dimensionless numbers, derived from fundamental physical principles, provide compact, scale-invariant representations of complex systems [18]. These dimensionless numbers compress crucial interactions, including forces, energies, and mechanisms, into dimensionless ratios that are physically interpretable and transferable across systems [17]. In sonochemical systems with highly nonlinear and multiparametric dynamics, such formulations might provide an opportunity for unifying the representation of different reactor configurations and operating conditions. Previous studies have attempted to incorporate dimensionless analysis into sonochemistry through the application of cavitation numbers [19,20]. These are dimensionless parameters that describe the system's tendency to undergo cavitation based on pressure differentials, vapour pressure, and fluid properties. Nevertheless, these efforts remain fragmented and lack systematic theoretical derivation or a complete set of dimensionless descriptors.

Unlike in fluid dynamics [21] or heat transfer [22], the field of sonochemistry lacks a broadly accepted set of dimensionless descriptors for consistent modelling. As a result, most existing modelling efforts rely on dimensional variables specific to particular experimental setups, which limits generalizability and interpretability. To address this gap, our previous work systematically derived a set of dimensionless *Π* terms specifically designed for sonochemical systems following Buckingham’s *Pi* theorem [23]. These variables capture key aspects of bubble dynamics and chemical reaction kinetics and were initially validated using regression models. The regression-based study confirmed that the *Π* terms effectively represent the initial reactor conditions in a dimensionless form and their link to the resulting chemical reactivity. However, due to the nonlinear and multiparametric nature of sonochemical responses [6,24], regression modelling required piecewise treatment of different parameter ranges, and validation on independent datasets indicated that it could reproduce overall trends but not provide accurate quantitative predictions [23].

Building on these findings, the present study develops a machine learning framework that integrates the validated dimensionless variables to achieve more accurate, robust, and generalizable prediction of sonochemical activity. Four representative sonochemical outputs were selected as modelling targets: sonochemiluminescence (SCL) intensity, SCL area, ultrasonic oxidation from iodide oxidation radicals (IORS), and ultrasonic oxidation from total reactive oxidative species (ROS), including both IORS and hydrogen peroxide (H_2_O_2_). The models built using dimensional and dimensionless input strategies in terms of predictive performance, extrapolation robustness, and interpretability were systematically compared. Furthermore, SHAP-based interpretability analysis was used to examine whether the model’s learned mechanisms aligned with known physical principles. Whereas the previous regression-based study primarily demonstrated the conceptual validity of the dimensionless *Π* terms, the present work extends this framework to nonlinear machine learning, enabling higher predictive accuracy and improved generalisability. In addition to predictive gains, the hybrid modelling strategy facilitates the visualisation and interpretation of underlying physical mechanisms, offering new insights into the complex dynamics of sonochemical processes.

## 2 Research method

### 2.1 Data collection

The dataset utilized in this study was generated from a series of systematically conducted experiments with a laboratory-built ultrasonic reaction system. Comprehensive details of the experimental procedures and raw measurement data have been reported in our previous work [24].

**Fig. 1:**
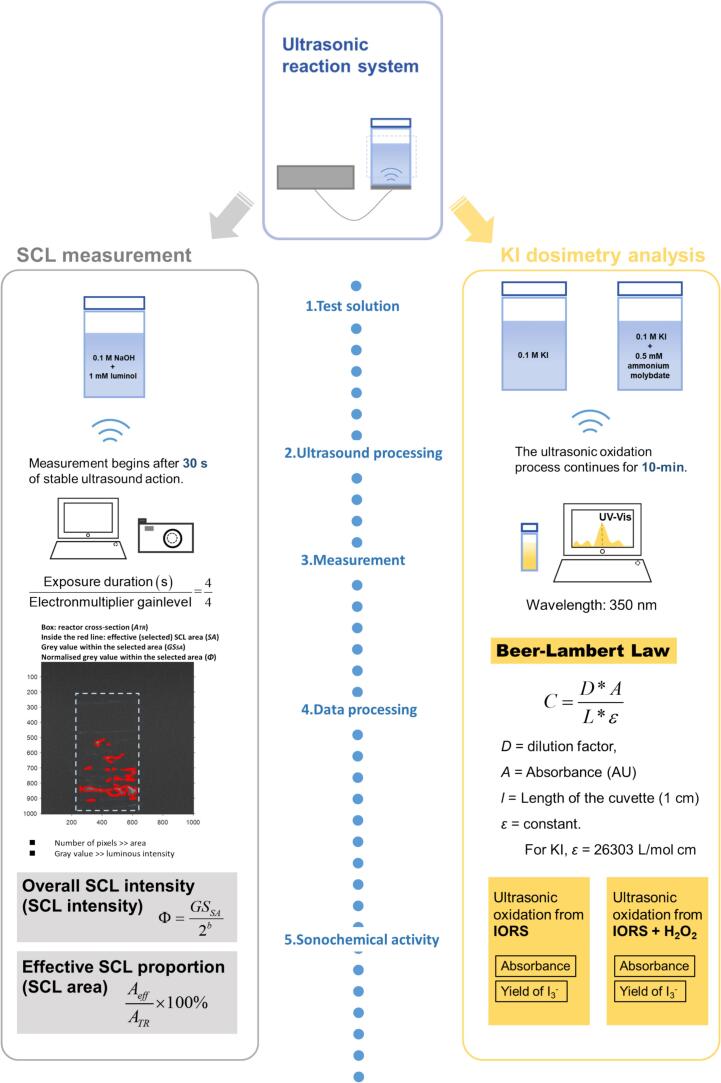
Schematic diagram for data collection.

Herein, 114 distinct operational configurations were investigated, each defined by three principal input variables: ultrasonic frequency (22–2000 kHz), load power (10–40 W), and liquid height (related to reactor volume: 200–400 mL). For each configuration, four indicators of sonochemical activity were measured (Fig. 1): SCL intensity, SCL area, oxidation caused primarily by iodide oxidation radicals (IORS), and total ROS-induced oxidation. Total ROS-induced oxidation was defined as the sum of IORS and H_2_O_2_. Since the KI dosimetry method is non-selective and cannot attribute the oxidation exclusively to hydroxyl radicals (·OH) [24], the term IORS denotes the ensemble of oxidising radicals capable of converting iodide to triiodide. The limited selectivity mainly results from side reactions, such as the formation of nitrous acid from dissolved nitrogen during sonication, which can also oxidise iodide ions [25]. In addition, the oxidation of iodide by H_2_O_2_ occurs only in the presence of a catalyst such as ammonium molybdate [26]. Therefore, when KI dosimetry is performed in the catalytic system, the total ROS-induced oxidation includes both IORS and H_2_O_2_.

Each test was repeated at least three times, and the average of the replicates was used in the final dataset for further analysis. The dataset consists of all original experimental findings, and no missing values were present.

### 2.2 Dimensionless variable set

The dimensionless variable set (Table 1) used in this study was adopted from our previous work [23], where it was systematically developed and validated. The symbols and definitions of the variables presented here are consistent with the earlier study and have not been modified. This allows us to directly assess how machine learning extends the predictive capability of the same physical descriptors.

**Table 1:** Set of dimensionless variables ‘*Π* terms’.

*Π* term	Expression	*Π* term	Expression
*Π1*	*ρ*_L_*H*_R_^3^*f*^2^/*σ*	*Π5*	*μ*_L_*H*_R_*f*/*σ*
*Π2*	*n*_w_/*n*_t_	*Π6*	*P*_∞_*H*_R_/*σ*
*Π3*	*c*/*H*_R_*f*	*Π7*	*C*_p_*T*_0_/(*H*_R_*f*)^2^
*Π4*	*I*_A_/*fσ*		

The dimensionless variable set was developed based on a theoretical review of bubble dynamics and chemical reaction kinetics relevant to sonochemical processes and formulated using the Buckingham’s *Pi* theorem [27]. Following the method of repeating variables [28], five base quantities (*f*, *H*_R_, *I*_A_, *T*_0_, and *n*_t_) were selected to span the relevant dimensional space. All additional relevant quantities were combined with these repeating variables to derive seven independent and physically meaningful dimensionless groups (*Π1*-*Π7*). These variables represent different aspects of sonochemical systems, such as bubble dynamics, vapor content, acoustic propagation, and thermal effect. These variables were previously validated and shown to effectively capture the relationship between initial operating conditions and various indicators of sonochemical activity. Definitions of all *Π* terms and associated physical parameters are provided in Supplementary information (SI, Appendix A, Tables S1 and S2).

The full derivation process and theoretical rationale for each term have been presented and validated in our previous publication [23]. In brief, this framework allows abstraction from system-specific dimensions and enables generalizable prediction across varying reactor conditions. The resulting set of dimensionless variables was used as input features for machine learning models, enabling direct comparisons with models based on raw dimensional parameters.

### 2.3 Dataset analysis

A structured dataset analysis was conducted before model construction to ensure reliable and generalizable model training. Machine learning models typically require well-scaled, outlier-free, and statistically representative input data to avoid biased learning and to improve generalisation [29]. Therefore, statistical and visualisation techniques were applied to the distribution of experimental variables, detect anomalies, and identify the need for data transformation or scaling. These insights informed subsequent preprocessing steps, including outlier removal, variable transformation (e.g., logarithmic scaling, square root transformation, min–max scaling, Z-score standardisation, and Box-Cox transformation), and normalisation. Such procedures are essential to ensure efficient learning and improve model performance [30].

Firstly, marginal histograms and joint scatter plots were used to visualise the distribution of individual operating parameters (frequency, power, and liquid height) and their association with sonochemical activity indicators (intensity and area of SCL, and ultrasonic oxidation from IORS (and H_2_O_2_)) across all tested experimental configurations. These visualisations are used to reveal the diversity and complexity of the experimental space in sonochemistry, thereby highlighting the need for structured modelling approaches capable of capturing nonlinear interactions and variable dependencies.

Box plots were used to examine the distribution of potential input and output variables to inform preprocessing strategy. Namely, input variables included the original operating parameters (ultrasonic frequency, power, and liquid height) and the seven dimensionless groups (*Π1*–*Π7*). Output variables consisted of four indicators of sonochemical activity: SCL intensity (*Φ*), active area proportion (*P*_eff_), IORS, and combined IORS + H_2_O_2_. Output variables exhibited noticeable right-skewed distributions and were transformed using a natural logarithmic function of the form log(1 + x) to reduce skewness and stabilise variance [31]. Among input variables, only ultrasonic frequency showed mild skewness, while others were approximately symmetric but varied in scale. Therefore, all input variables were standardised using Z-score normalisation to ensure uniform scaling and prevent dominance of variables with larger magnitudes during model training [32].

Pearson correlation coefficient matrices (*r*) were calculated to assess the linear relationships among all input and output variables, including both dimensional and dimensionless forms. For dimensional variables, correlations were calculated directly based on the collected dataset (experimental parameters and results).

For the dimensionless variables, a synthetic parameter matrix (Table. 2) including 1875 combinations of different operating conditions was constructed to enhance the robustness of correlation analysis. Based on the matrix, the corresponding dimensionless input variables (*Π1–Π7*) were calculated, and their Pearson correlations analysed. Importantly, this analysis was restricted to input variables and did not involve output indicators. Furthermore, Variance Inflation Factor (VIF) analysis was carried out on the dimensionless inputs derived from the parametric matrix to assess the presence of multicollinearity [13]. Pearson correlation and VIF analysis were conducted to assess inter-variable dependencies and confirm the independence of all seven dimensionless inputs, ensuring their suitability for subsequent modelling.

**Table 2:** Parameter matrix for dimensionless variable independence testing.

Parameters	Setting values
Ultrasonic frequency (kHz)	22, 44, 200, 500, 700
Load power (W)	10, 20, 30, 40, 50
Reactor volume (ml)	200, 300, 400, 500, 600
Reactor inner diameter (cm)	5, 6.7, 10
Initial temperature (K)	293, 303, 313, 323, 333

Based on the analyses described above, the final variable set used for model development was determined through feature selection and transformation. These analyses included box plot inspection to identify distribution patterns and outliers, logarithmic transformation applied to output variables for variance stabilisation, and standardisation applied to all input variables to ensure consistent scaling. Pearson correlation and VIF analyses were performed to examine dependencies among the input variables. The assessment was primarily conducted on the dimensionless input variables, while the dimensional inputs were also considered in the correlation analysis. This process ensured that the selected input features were sufficiently independent for use in modelling.

### 2.4 Model construction and evaluation

**Fig. 2:**
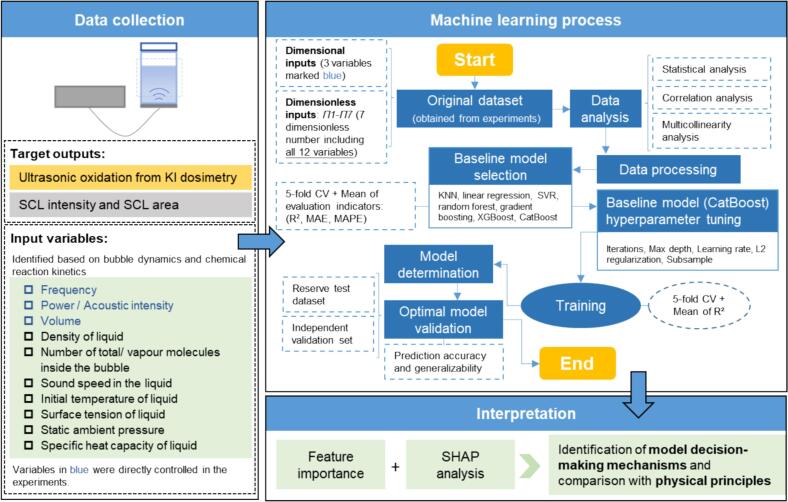
Schematic overview of the modelling workflow in this work.

The aim of implementing machine learning models in this study was to predict sonochemical responses, including SCL and ultrasonic oxidation. To achieve this, the models were trained using either dimensional inputs (e.g., ultrasonic frequency, load power, liquid height) or (dimensionless inputs (*Π1–Π7*), in order to capture their respective nonlinear relationships with sonochemical responses. This task was formulated as a supervised learning problem, where the objective is to learn a functional relationship between known inputs and experimentally observed outputs [33]. An overview of the modelling workflow, including variable selection, data processing, model training, and interpretation, is provided in Fig. 2.

To identify the most suitable model, several representative supervised regression algorithms were implemented and compared: KNN, linear regression, SVR, random forest, gradient boosting, XGBoost, and CatBoost. The modelling process comprised three major stages: (1) selection and construction of candidate models; (2) evaluation through five-fold cross-validation (5-fold CV) using statistical metrics: coefficient of determination (R^2^), mean absolute error (MAE), and mean absolute percentage error (MAPE); and (3) hyperparameter optimization of the selected model to optimal predictive accuracy.

All machine learning tasks were implemented in Python 3.12 using CatBoost for model training, scikit-learn for data preprocessing and evaluation, and SHAP for feature interpretability. Data handling and visualisation were performed using pandas, NumPy, and matplotlib. All computations were conducted on a standard Windows 10 laptop (Intel i5 CPU, 16 GB RAM) without GPU acceleration.

#### 2.4.1 Selection of machine learning algorithms

Differences among machine learning models are not limited to structural complexity; they also involve trade-offs between bias and variance, sensitivity to the size of the training dataset, and robustness to noise and distributional shifts [34]. Since model performance can vary significantly across different datasets and problem settings, it is often impossible to determine the best-performing algorithm in advance [35]. Therefore, it is necessary to make comparisons that are adapted to the dataset and the prediction task.

Seven representative supervised regression algorithms were initially selected for preliminary screening to assess their ability to predict sonochemical activity under various ultrasonic operating conditions. These models cover a broad range of learning rules, including instance-based (KNN), parametric (linear regression), kernel-based (SVR), and ensemble methods. The ensemble models include random forest (bagging) and three boosting models—Gradient Boosting, XGBoost, and CatBoost. The selection of these seven algorithms was deliberate rather than arbitrary, aiming to represent the major paradigms of supervised regression, including linear, kernel-based, instance-based, and ensemble learning methods. Model characteristics, data properties, and the requirements of the prediction task guided the choice. A detailed rationale for each algorithm is provided in Appendix B.

#### 2.4.2 Model evaluation

A 5-fold CV approach was employed for all alternative model evaluations. The dataset was randomly divided into five equal subsets. In each fold, four subsets were used for training and one for validation, repeated five times to ensure each subset served as the validation set once. This strategy was used to mitigate the variance caused by data partitioning and reduces the risk of overfitting due to a single train-test split [34].

Three performance metrics were used to evaluate model accuracy: R^2^, MAE, and MAPE (Table 3). In the Table 3, *n* denotes the number of samples, $y_{i}$ signifies the actual value of the observation, and ${\hat{y}}_{i}$ represents the forecast value of the model, and $\overset{¯}{y}$ indicates the mean of the actual values.

**Table 3:** Model performance evaluation metrics.

Evaluation metrics	Mathematical expression
R^2^	$\frac{\sum_{i=1}^{n}{\left({\hat{y}}_{i}-\overset{¯}{y}\right)}^{2}}{\sum_{i=1}^{n}{\left(y_{i}-\overset{¯}{y}\right)}^{2}}$(1)
MAE	$\frac{1}{n}\sum_{i=1}^{n}\left|y_{i}-{\hat{y}}_{i}\right|$(2)
MAPE	$\frac{100}{n}\sum_{i=1}^{n}\left|\frac{y_{i}-{\hat{y}}_{i}}{y_{i}}\right|$(3)

R^2^ reflects the proportion of variance in the output that is captured by the model and serves as a global goodness-of-fit indicator. MAE quantifies the value of absolute prediction errors. MAPE expresses error in relative terms, facilitating interpretability across different output scales.

The same cross-validation strategy and evaluation metrics were applied during hyperparameter tuning (see Section 2.4.3). The optimal parameter configuration was identified based on the average performance across folds and was subsequently used to reconfigure the model.

#### 2.4.3 Model tuning

Hyperparameter tuning is critical in improving the model's predictive performance and generalizability. Hyperparameter configurations govern the model's complexity, learning behaviour, and regularization strength.

In this study, grid search was used as the hyperparameter optimization strategy. For the final selected model (CatBoost), the parameter grid was defined based on recommendations from related published studies [36] and further refined according to the specific size and characteristics of the dataset used in this work (Table 4). The search was performed using a 5-fold CV, with the average coefficient of R^2^ value across folds used as the primary selection criteria.

**Table 4:** Hyperparameter range for grid search.

Hyperparameter	Range
Iterations	100, 200, 300
Max depth	4, 6, 8
Learning rate	0.01, 0.03, 0.05, 0.1
L2 regularization	1, 3, 5
Subsample	0.8, 1.0

Once the best-performing parameter set was identified, the selected model (CatBoost) was retrained using the entire training portion of the dataset. A new train-validation split was then applied to the full dataset. Specifically, 80 % of the data was used to fit the model with the optimal hyperparameters, while the remaining 20 % was reserved to evaluate its predictive capability.

After hyperparameter tuning, the R^2^ was used as the primary evaluation criterion, as it directly quantifies the model’s generalisation ability and facilitates the detection of overfitting through comparison of training and test performance. Meanwhile, error-based metrics (MAE and MAPE) were already evaluated during the initial model screening stage and were therefore not repeated to avoid redundancy and maintain clarity in performance reporting.

#### 2.4.4 Final model evaluation

To assess the prediction and generalization performance of the final models, a hold-out validation approach was adopted based on the reserved validation set. Model performance on the test set was quantified using R^2^.

Additionally, an external validation step was conducted using experimental data extracted from previously published sonochemical studies [[37], [38], [39], [40]]. The optimized model’s predictions were compared against these independent data to assess its generalizability beyond the current dataset.

### 2.5 Feature importance and SHAP-based interpretation

To enhance the interpretability of the final model and strengthen its practical relevance in sonochemistry, two complementary methods were used to analyse the influence of input variables: feature importance analysis and SHAP analysis.

Feature importance scores were extracted directly from the trained CatBoost model to assess each variable’s relative impact globally. SHAP values were computed based on the Shapley value framework from cooperative game theory (Eq. 4) [41], which attributes a fair contribution to each feature in the prediction process, and expresses the influence marginal effect of that feature [15].

(4) $$\varphi_{i}\left(x\right)=\sum_{Q\subseteq S\backslash \left[\text{i}\right]}\frac{\left|Q\right|!\left(\left|S\right|-\left|Q\right|-1\right)!}{\left|S\right|!}\left(\Delta_{Q\cup \left[i\right]}\left(x\right)-\Delta_{Q}\left(x\right)\right)$$

Where *φ_i_*(*x*) represents the Shapley value of the feature *i*, *S* is the full set of features, and *Q* is a subset of *S* that does not include feature *i*. △_Q_(*x*) denotes the model's output when only the features in subset *Q* are considered. The difference, △_Q∪[i]_ (*x*) − △_Q_(*x*), quantifies the individual contribution of feature *I* to the prediction for instance *x*.

To obtain more refined and locally accurate insights, the SHAP analysis was conducted using the TreeExplainer algorithm, which is optimised explicitly for tree-based ensemble models such as CatBoost, XGBoost and Gradient boosting [42].

## 3 Results and discussion

### 3.1 Dataset analysis

#### 3.1.1 Preliminary analysis of sonochemical single parametric response

**Fig. 3:**
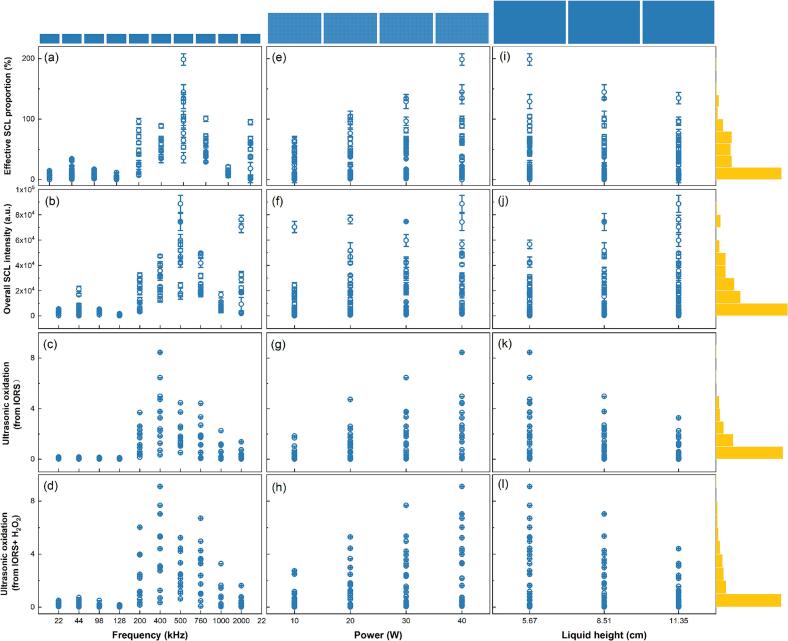
Marginal histograms of operating parameters and sonochemical activity.

Distribution relationships between the dimensional input variables (frequency, power, liquid height) and the output variables (SCL intensity and area, and ultrasonic oxidation (from IORS + (H_2_O_2_)) are not uniform (Fig. 3). Specifically, the influence of each input variable on the outputs is neither linear nor monotonic; rather, it involves multidimensional and nonlinear variations. This makes it difficult to fully explain output variations based on a single variable [43]. For example, the peak of ultrasonic oxidation is observed around 500 kHz (Fig. 3(c) and (d)), while the SCL-based activity (Fig. 3(a) and (b)) reaches its maxima at both 44 kHz (low frequency) and 400 kHz (intermediate frequency). These observations indicate that a simple linear trend cannot explain frequency-sensitive cavitation kinetics. In Fig. 3(e–h), an overall increase in sonochemical activity is observed with increasing power; however, the relationship is nonlinear and tends to plateau at higher input levels (e.g., 40 W in this study). In particular, an apparent saturation behaviour is seen in Fig. 3(f), where SCL intensity is off beyond 30 W, indicating the presence of a threshold effect [7]. Moreover, liquid height (Fig. 3(i–l)) is intrinsically coupled with the power threshold effect since volume (reflected by liquid height) and power jointly determine the spatial distribution of energy in the system. The interplay between input power and liquid height thus governs whether the effective power density reaches [38], exceeds, or remains below the cavitation threshold—ultimately controlling the onset and sustainability of sonochemical activity. Therefore, the combined actions between key parameters should be systematically considered when optimising an ultrasonic reaction system.

#### 3.1.2 Statistical analysis of input data

**Fig. 4:**
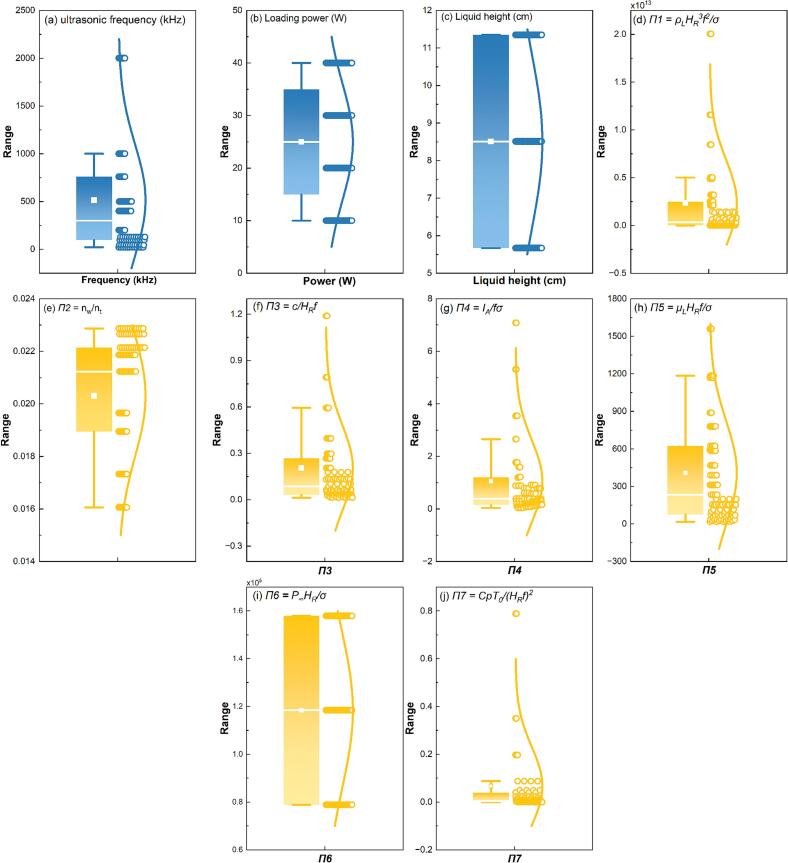
Boxplot of input variables: dimensional variable (a–c) and dimensionless variable (d–j).

**Fig. 5:**
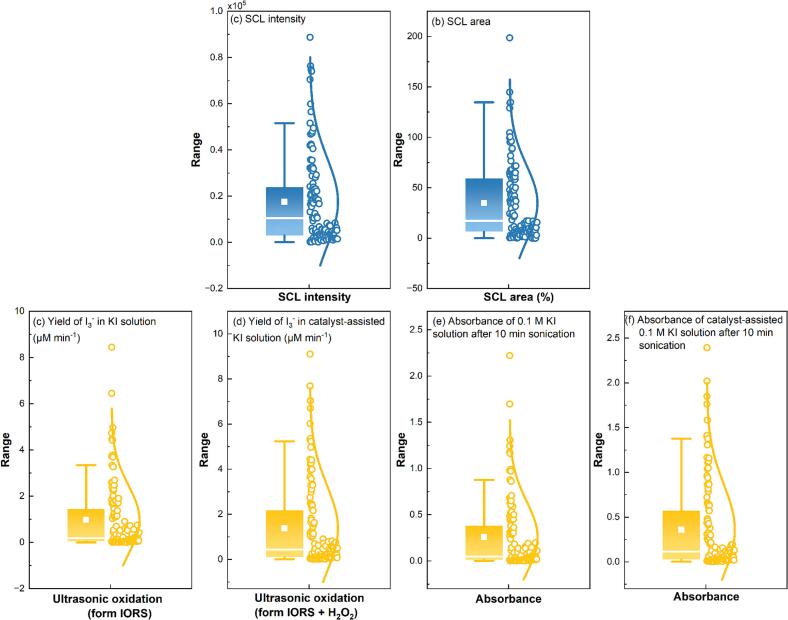
Boxplot of output variables: SCL intensity (a), SCL area (b), ultrasonic oxidation (from IORS, dimension (c) and dimensionless (e)), and ultrasonic oxidation (from IORS + H_2_O_2_, dimension (d) and dimensionless (f)).

Boxplot analysis of the input and output data – Fig. 4, Fig. 5 respectively was used to guide necessary data preprocessing [44].

Fig. 4 shows the distribution characteristics of all input variables, including both operational parameters (Fig. 4 (a–c)) and dimensionless numbers’ group (Fig. 4 (d–j)) that reflect the initial setting of the system. The frequency (Fig. 4 (a)) exhibits a noticeable right-skewed distribution, with most data points concentrated in the lower to medium range (e.g., <760 kHz), with a few values at 2000 kHz. The wide dynamic range and skew indicate that normalisation is necessary to prevent dominant influence during training. The experimental settings of power (Fig. 4(b)) and liquid height (Fig. 4(c)) were generally symmetric. Despite the absence of noticeable skewness or extreme outliers, the numerical ranges and units of power and height differ significantly from each other. To ensure comparability and equal weighting of all dimensional inputs in the machine learning models, all three variables were standardised using Z-score normalisation prior to training. This step improves robustness by preventing variables with larger magnitudes from disproportionately influencing model learning.

Fig. 4 (d–j) shows the distribution of the dimensionless group (values of *Π1*–*Π7* in the dataset) encompassing various sonochemistry-involved physical quantities. These variables demonstrate the interrelated effects of ultrasonic operation settings, the reaction solution's properties and the reactor's geometric configuration, resulting in more intricate distribution patterns than the raw experimental inputs [23]. Specifically, *Π1* and *Π5* demonstrate the broadest distributions, encompassing multiple orders of magnitude and featuring notable outliers. *Π2* and *Π6* exhibit comparatively compact distributions due to the estimation [23] and the experimental mode. Nonetheless, *Π2* has a pronounced upper-bound clustering. *Π3*, *Π4* and *Π7* exhibit mild skewness. While dimensionless transformation removes unit inconsistencies, the variables differ significantly in scale and distribution. Therefore, additional standardisation was required prior to model training.

Therefore, all input variables were normalised by a Z-score transformation (centred to 0 and the standard deviation scaled to 1) prior to model training [32].

#### 3.1.3 Statistical analysis of output data

The dataset includes multiple target variables representing sonochemical activity. These outputs fall into two main categories: SCL (Fig. 5 (a) and (b)) and ultrasonic oxidation measured via KI dosimetry (Fig. 5 (c–f)).

The SCL-related outputs, e.g. SCL intensity and SCL area, are already dimensionless [23], having been derived from image-based quantification [24,45]. These outputs are used under two modelling strategies: one based on raw experimental parameters (dimensional) and the other using dimensionless input variables (*Π1*-*Π7*). However, it is important to note that SCL data are influenced by the quality of the captured images, introducing potential variability. Therefore, only data collected under 22–1000 kHz conditions were included for model training involving SCL outputs. This dataset was selected based on quality filtering discussed in the Supplementary information (SI, Appendix C). For ultrasonic oxidation, two formats of experimental findings are used: the quantitative I_3_^-^ yield (with dimensions calculated from Beer-Lambert law) and the absorbance values (dimensionless, obtained via UV–Vis). To ensure consistency with the respective modelling strategies, yield data were used in models with dimensional inputs, while absorbance values were used in models using dimensionless inputs. As additional factors or steps do not influence KI dosimetry, the entire dataset spanning 22–2000 kHz was included in model training.

It is important to note that the dataset used in this work is entirely derived from experimental results, with no artificial data synthesis. Therefore, except for specific samples discarded due to quality issues (e.g., SCL analysis), no outlier removal was performed. Retaining extreme values is important, as they may represent attractive sonochemical responses under optimal parameter combinations. This process enables a wide range of parameter space while maintaining the diversity and richness of the experimental data.

As shown in Fig. 5, all output variables exhibit strong positive skewness, characterized by long-tail distributions and extreme values. For instance, both SCL intensity (Fig. 5(a)) and SCL area (Fig. 5(b)) show high variability and outliers, and outputs from ultrasonic oxidation, including I_3_^-^ yield (Fig. 5 (c–d)) and absorbance values (Fig. 5 (e–f)), also display substantial deviation from normality. These distributional properties suggest potential instability and bias in direct regression modelling using raw output values [46]. To address this, a natural logarithmic transformation of the form log(1 + x) (as known log1p function) was initially applied for all output variables. This transformation is widely recognized for compressing dynamic range, reducing skewness, stabilizing variance, and improving predictive performance and generalizability in machine learning models [31,47].

#### 3.1.4 Correlation and multicollinearity analysis of variables

**Fig. 6:**
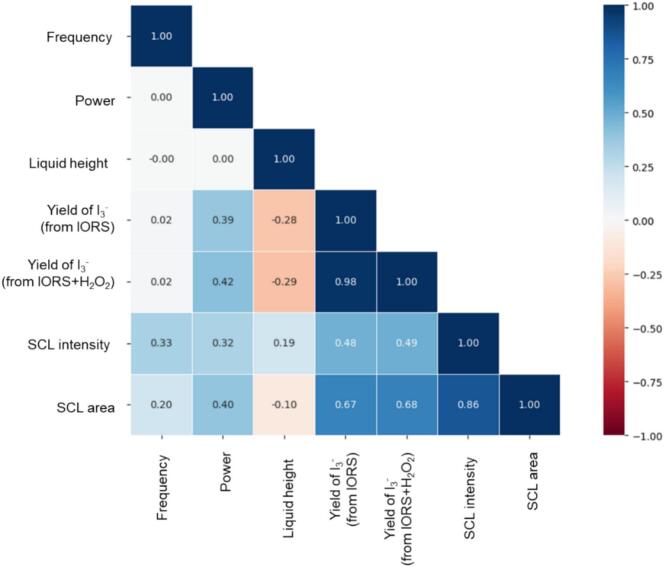
Pearson correlation of dimensional input variables and target outputs based on raw experimental results.

**Fig. 7:**
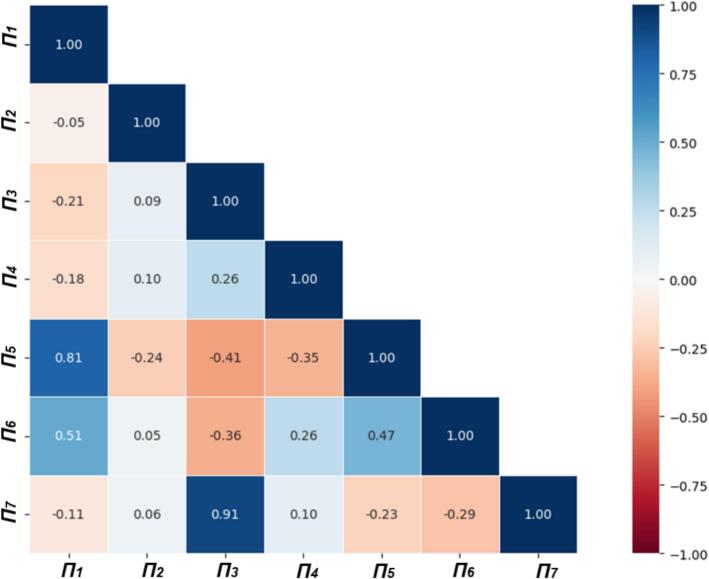
Pearson correlation of dimensionless input variables based on the synthetic parameter matrix.

The detailed linear relationships among variables were assessed using the Pearson correlation coefficient for dimensional and dimensionless variables [48], Fig. 6, Fig. 7, respectively. The coefficient ranges from −1.00 to + 1.00, indicating the strength and direction of the linear relationship between the two variables. A coefficient closer to + 1.00 denotes a strong positive correlation, whereas a value closer to −1.00 indicates a strong negative correlation. A coefficient near 0 suggests little to no linear relationship between the variables [48,49].

As shown in Fig. 6, the correlations among frequency, power, and liquid height are essentially zero, indicating that these operational parameters are statistically independent. This independence supports their use as non-interfering input features, providing higher-dimensional and uncorrelated information for machine learning.

The four output variables, SCL intensity, SCL area, yield of I_3_^-^ (from IORS), and yield of I_3_^-^ (from IORS + H_2_O_2_), demonstrate differing levels of relationship with the input features. Power displays a positive correlation with all four sonochemical activities (*r*＞0). Liquid height negatively correlates with ultrasonic oxidation (*r*＜0) and SCL area (*r* = -0.1＜0), while positively correlates with SCL intensity (*r* = 0.19＞0). Notably, the very low correlation coefficient (*r* = 0.02) between frequency and the I_3_^-^ yield (from IORS (+ H_2_O_2_)) highlights the complex role of frequency in the sonochemical mechanism.

To improve the robustness of correlation analysis, the Pearson correlation coefficients among the seven dimensionless input variables (Fig. 7) were calculated using a synthetic parameter matrix composed of 1875 combinations of operating conditions (Table 2). Strong correlations between *Π3* and *Π7* (*r* = 0.91) and *Π1* and *Π5* (*r* = 0.81) were found in Fig. 7, suggesting that there may be information redundancy between these variable pairs. Despite this, most other variable groups exhibit correlation coefficients within the range of −0.5 to + 0.5, suggesting an acceptable level of independence for multivariate modelling [50].

To further assess multicollinearity among the dimensionless input variables (*Π1*-*Π7*), variance inflation factors (VIF) were calculated based on the values of the dimensionless numbers generated from the synthetic parameter matrix (Table 2). The results are summarised in Table 5. This analysis evaluates the extent to which each Π-term can be linearly predicted from a combination of the others, thereby revealing potential redundancy among the inputs. While *Π3* (VIF = 6.33) and *Π7* (VIF = 6.10) demonstrate medium degree multicollinearity, their values remain below the usual critical threshold for significant collinearity (VIF < 10), suggesting their influence on modelling is acceptable [51] and implying that their inclusion will not unduly bias model training. All other VIF values are under 5, confirming low interdependence among the remaining variables.

**Table 5:** Variance inflation factor of the independent variables.

The dimensionless variable	Variance inflation factor (VIF)
*Π1*	2.868
*Π2*	0.861
*Π3*	6.33
*Π4*	1.844
*Π5*	3.19
*Π6*	1.73
*Π7*	6.11

Given that the observed correlation and multicollinearity were within acceptable limits, and considering the theoretical grounding of each variable, all seven dimensionless groups were retained to balance statistical robustness with physical interpretability. Significantly, these dimensionless variables are not arbitrary statistical constructs but are derived from fundamental physical mechanisms within the sonochemical system [23]. Consequently, deleting any of the variables only due to statistical duplication could negatively impact the model's physical interpretability and ability to be applied across broad experimental conditions. Following the view of mechanistic completeness in physics-guided machine learning [52], this decision supports the development of accurate, physically meaningful, and generalisable models.

### 3.2 Baseline (machine learning) model selection

**Fig. 8:**
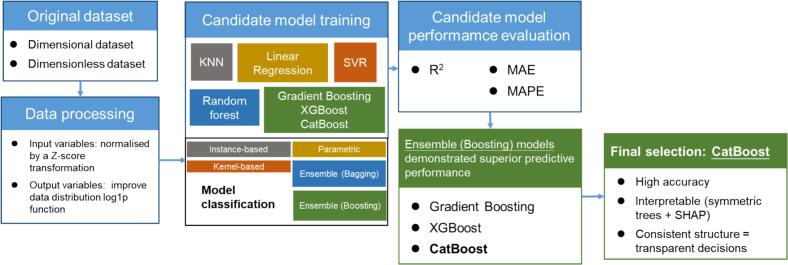
Workflow of model selection process.

To investigate the effectiveness of machine learning in predicting sonochemical activity under varying operational conditions, the performance of seven supervised regression algorithms, e.g., KNN, linear regression, SVR, random forest, gradient boosting, XGBoost, and CatBoost, were systematically assessed. Fig. 8 shows the model selection process, with CatBoost selected as the final model.

Among all candidate models, boosting models (i.e., gradient boosting, XGBoost, CatBoost) consistently achieved attractive performance across various sonochemical tasks (see detailed comparisons in Appendix D). Their robustness and capacity for modelling complex nonlinearity make them strongly considered for baseline model selection [53,54].

As an experimental domain, model interpretability is critical for bridging data-driven predictions with mechanistic understanding in sonochemistry [52,55]. Both CatBoost and XGBoost exhibit strong predictive performance and support SHAP-based explanation frameworks. A key advantage of CatBoost lies in its use of oblivious (symmetric) tree structures, where the same split condition is applied at each level of the tree [54]. This produces uniformly deep trees with consistent branching logic, improving decision paths' transparency and traceability. Compared to traditional asymmetric decision trees (e.g., CART used in XGBoost and standard gradient boosting models), symmetric trees enable a more structured analysis of feature contributions, leading to clearer and more robust [56]. Consequently, CatBoost was selected as the final baseline model, balancing predictive accuracy with interpretability.

### 3.3 Predictive performance of CatBoost with dimensional inputs

To evaluate the predictive performance of the CatBoost algorithm using dimensional inputs, models were trained with three operational parameters (i.e., frequency, power, and liquid height). Preprocessing followed the procedures described in section 2.3, including standardisation of inputs and logarithmic transformation of outputs. Compared to regression-based approaches, which required piecewise treatment of parameter ranges due to the nonlinear and multiparametric nature of sonochemical responses, the machine learning framework was able to capture these nonlinearities directly, without prior segmentation [13,49]. This allowed the model to handle the full parameter space in a unified manner, reducing the need for subjective segmentation decisions and thereby improving prediction accuracy and efficiency.

**Fig. 9:**
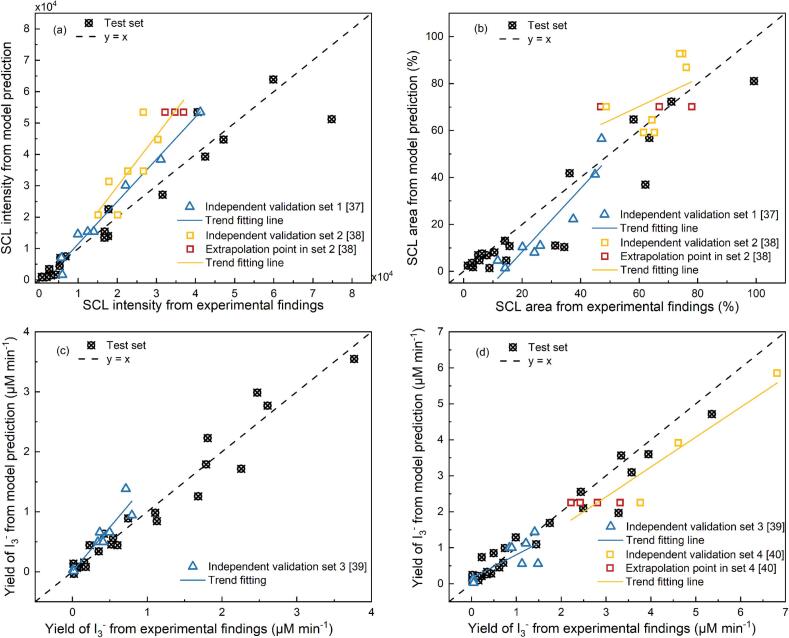
Model predictions vs. experimental results using CatBoost with dimensional inputs: SCL intensity (a), SCL area (b), Yield of I_3_^-^ from IORS (c), and Yield of I_3_^-^ from IORS + H_2_O_2_ (d).

The optimal hyperparameter sets and the corresponding model performance for each prediction target are summarised in Table 6. The CatBoost models displayed strong predictive accuracy for all target variables, with cross-validated mean R^2^ values ranging from 0.87 to 0.96. The minor standard deviations (particularly in ≤ 0.04) indicate consistent model performance across different folds, suggesting robustness and stability against data variability. Moreover, the final models achieved high R^2^ values on the reserved test sets (0.86–0.95), verifying generalisability and indicating that no obvious overfitting occurred. This can be attributed to the appropriateness of the selected hyperparameter configurations. Additionally, the CatBoost algorithm incorporates techniques such as symmetric tree structures, ordered boosting, and L2 regularisation, which help effectively reduce overfitting [36,54]. This result was visualised in Fig. 9; most test samples (black symbols) are closely distributed around the ideal line (y = x).

**Table 6:** Optimized hyperparameters and model performance for target variables.

**Prediction Target**	**SCL intensity**	**SCL area**	**Ultrasonic oxidation from IORS**	**Ultrasonic oxidation from IORS + H_2_O_2_**
Iterations	200	300	300	300
Max depth	4	4	4	4
Learning Rate	0.1	0.1	0.05	0.1
L2 Regularization	3	1	1	1
Subsample	0.8	1	1	0.8
Model performance in training set (mean R^2^)	0.87 ± 0.03	0.92 ± 0.04	0.95 ± 0.02	0.96 ± 0.01
Model performance in whole training set (R^2^)	0.98	0.99	0.99	0.99
Model performance in test set (R^2^)	0.91	0.86	0.95	0.93

To evaluate the generalizability of the trained CatBoost models beyond the training domain, their predictive capacity was also validated on independent experimental data collected from publication studies (Fig. 9). Overall, most validation points (triangles and squares) generally align with the fitted trend lines, suggesting that the models capture the trends of SCL and I_3_^-^ yields under varying conditions outside the original training dataset. It is also worth noting that chemical dosimetry indicators (IORS and IORS + H_2_O_2_) were predicted more accurately than SCL descriptors (intensity and area). This is consistent with the test set results in Table 6, where the chemical targets achieved higher R^2^ values (0.95 and 0.93) compared to the optical targets (0.91 and 0.88). Moreover, in the independent validation sets, chemical indicators exhibited predictions closer to the ideal line. This difference likely arises from the higher quantitative reliability of chemical dosimetry, whereas SCL measurements are more susceptible to experimental noise and the sensitivity of optical setup and image-processing procedures [23].

Validation set 1, set 2 and set 3 were obtained from cylindrical reactors with the same inner diameter as the training set but included operating conditions not present in the training data, such as different initial temperatures [37], frequency [37,39], powers and volumes [38]. Despite the omission of initial temperature as an input variable, the models still reproduced the trends of SCL intensity and SCL area reasonably well. It should be noted, however, that this agreement was obtained under conditions where the temperature difference was slight (25 °C vs. 20 °C), so the predictions remain of indicative value even without explicitly including temperature as an input. However, validation set 2 contained much higher powers (up to 100 W compared to a maximum of 40 W in the training set). In this extrapolation region, the model failed to capture the correct trend and produced plateau-like predictions (Fig. 9 (a) and (b)), reflecting its inability to generalise far beyond the training range. Validation set 4 introduced even greater challenges (Fig. 9 (d)), as it involved a reactor with a different inner diameter, unobserved liquid volumes (100 mL and 150 mL), and higher powers (60 W). These combined differences and their potential effects on acoustic field distribution, wave reflection, and diffraction [6], prevented the model from effectively internalising the relationships among input variables. As a result, larger predictive deviations from the reference line (y = x) and predicted plateaus were observed. These results indicate that while dimensional-input CatBoost models generalise well within or near the training domain, their performance deteriorates sharply when extrapolated to substantially different operational ranges or reactor configurations. This deterioration may be attributed to insufficient data coverage in the extrapolation region, which prevents the model from learning relevant patterns. In addition, ensemble models tend to produce conservative predictions under data sparsity [57].

In summary, the dimensional-input CatBoost models generalise well within and near the training domain but become less reliable when extrapolated to substantially different operational ranges or reactor configurations for sonochemical activities (SCL and ultrasonic oxidation). Caution is therefore required when applying the models beyond the trained space, as predictive accuracy may deteriorate due to limited data coverage and unlearned patterns. These findings underscore that predictive models based on dimensional inputs place higher demands on dataset coverage and the inclusion of physicochemical descriptors, which are essential for improving their generalisability and applicability in sonochemical systems.

### 3.4 Predictive performance of CatBoost with dimensionless inputs

To maintain consistency with the dimensional modelling pipeline, we initially adopted the same preprocessing strategy for the dimensionless-input models: input variables were standardized using Z-score, and the output variable was transformed using the natural logarithm function log(1 + x). However, preliminary results indicated a substantial drop in predictive performance compared to the dimensional-input models. In particular, the built models displayed limited generalization capability when applied to the independent validation set. The discrepancy can be linked to the characteristics of the dimensionless variables. These input variables are defined via theoretically assessed nonlinear combinations of physical parameters [23], which inherently incorporate complex nonlinear interactions and dynamic scaling relationships [58]. Applying an extra logarithmic transformation on the output might accidentally change the fundamental functional relationships between inputs and objective values, reducing the model's capacity to learn substantial patterns. Therefore, while maintaining standardized inputs, models using the dimensionless input strategy were retrained with the raw (non-log-transformed) dimensionless output values. This adjustment significantly improved the predictive accuracy and generalizability of the model. It highlights the importance of aligning the output transformation strategy with the structural characteristics of the input feature space [59].

Following the adjustment of the output preprocessing strategy, we trained CatBoost models using standardized dimensionless inputs and raw output values. Optimized CatBoost models exhibited robust predictive performance across all target variables (Table 7). For all four targets (SCL and ultrasonic oxidation), mean R^2^ scores from 5-fold CV ranged between 0.81 and 0.86, with relatively small standard deviations (e.g., ±0.04 for SCL intensity, ±0.07 for SCL area), indicating good model stability and low sensitivity to sample splits. When retrained on the entire training dataset, the final models achieved R^2^ values between 0.87 and 0.95 on the reserved test set, suggesting strong generalization capability.

**Table 7:** Optimized hyperparameters and model performance for target variables.

**Prediction Target**	**SCL intensity**	**SCL area**	**Ultrasonic oxidation from IORS**	**Ultrasonic oxidation from IORS + H_2_O_2_**
Iterations	300	300	300	300
Max depth	4	4	4	4
Learning Rate	0.1	0.1	0.1	0.1
L2 Regularization	1	1	1	1
Subsample	0.8	0.8	0.8	1
Model performance in training set (mean R^2^)	0.83 ± 0.04	0.86 ± 0.07	0.84 ± 0.09	0.81 ± 0.12
Model performance in whole training set (R^2^)	0.99	0.99	0.99	0.99
Model performance in test set (R^2^)	0.92	0.87	0.95	0.95

In addition, to benchmark against our previous regression-based framework, Table 8 compares the predictive accuracy of regression and machine learning models under the dimensionless strategy. The regression-based models achieved piecewise R^2^ values between 0.67 and 0.90 across different frequency ranges and prediction targets [23]. These values were calculated on the full dataset, requiring segmentation into separate parameter ranges due to the nonlinear and multiparametric nature of sonochemical responses. By contrast, the machine learning models established here captured the full parameter space in a unified manner and consistently achieved higher R^2^ values on independent reserved test sets. While the datasets used for R^2^ calculation are not identical, the comparison still illustrates the clear advantage of the machine learning framework: avoiding subjective segmentation and delivering superior predictive accuracy and generalisability.

**Table 8:** Comparison of R^2^ values from regression models (full dataset) [23] and machine learning models (reserved test data) for predicting sonochemical activity.

	**Regression model (piecewise R^2^)**	**Machine learning model (test set R^2^)**
**SCL intensity**	0.71 (22 – 128 kHz); 0.83 (200 – 760 kHz)	0.92 (22 – 1000 kHz)
**SCL area**	0.71 (22 – 128 kHz); 0.76 (200 – 760 kHz)	0.87 (22 – 1000 kHz)
**Ultrasonic oxidation from IORS**	0.67 (22 – 128 kHz); 0.86 (200 – 2000 kHz)	0.95 (22 – 2000 kHz)
**Ultrasonic oxidation from IORS + H_2_O_2_**	0.87 (22 – 128 kHz); 0.90 (200 – 2000 kHz)	0.95 (22 – 2000 kHz)
**Overall R^2^**	0.67 – 0.90	0.87 – 0.95

Beyond outperforming regression in predictive accuracy, the machine learning models trained with dimensionless inputs also exhibited notable consistency in their internal configurations. Under the dimensionless input strategy, the optimised hyperparameters for all four prediction targets converged to nearly identical configurations (Table 7). The observed consistency indicates that the dimensionless variables develop a more uniform and organised feature space, allowing for different objectives to demonstrate comparable learning complexity. Such alignment in model structure requirements indicates that the nonlinear relationships between inputs and outputs are more uniformly captured across tasks. The reduced sensitivity to hyperparameter tuning improves model deployability and replicability across different tasks. This outcome lowers the cost of model adaptation and enhances transferability [60]. Furthermore, this phenomenon suggests shared physical mechanisms or similar nonlinear coupling structures among different sonochemical responses when expressed in dimensionless form. This observation aligns with the principle of dimensional analysis, which aims to unify the representation of complex physical processes [58]. It also supports the theory that physics-guided feature engineering can improve model robustness and interpretability [52].

**Fig. 10:**
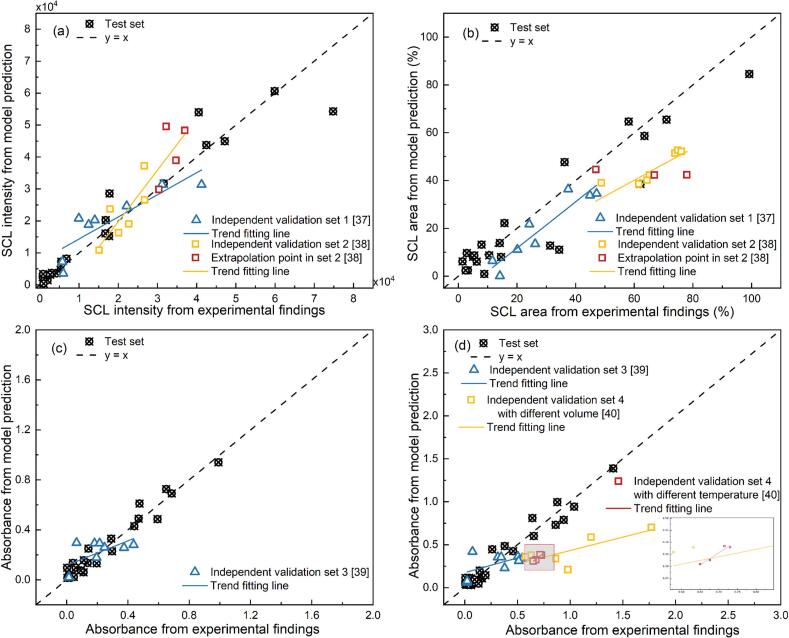
Model predictions vs. experimental results using CatBoost with dimensionless inputs: SCL intensity (a), SCL area (b), Absorbance from KI solution after 10 min sonication (c), and Absorbance in catalyst-assisted KI solution after 10 min sonication (d).

The CatBoost models trained using dimensionless inputs achieved strong performance in predicting the four target outputs across both the test set and several external validation datasets (Fig. 10). When extrapolated to external datasets, the model retained varying degrees of generalization capacity. This capability contrasts with the previous regression-based models, which could only reproduce overall trends across piecewise parameter ranges but required dataset-specific correction terms when applied to independent validation sets [23]. As a result, the regression models could not provide universally valid quantitative predictions, whereas the machine learning framework established here delivers direct and reliable quantitative references across internal and external datasets.

The validation set 1 [37], which employed a previously unseen ultrasonic frequency (300 kHz) and a higher initial temperature (25°C), yielded prediction points closely clustered along the fitted trend line, indicating effective model generalization to moderate physical variations (blue triangle in Fig. 10 (a) and (b)). The external validation set 2 introduced a broader power range (20–100 W) and larger liquid volumes (up to 500 mL), which were beyond the scope of the training data [38]. The reaction volume and ultrasonic power changes can significantly affect the sound field distribution and cavitation effect [61,62]. In contrast to the dimensional-input model, where the set 2 points exhibit a prediction plateau (red square in Fig. 9 (a) and (b)), the dimensionless-input model successfully decreased this effect (Fig. 10 (a)). Rather than stagnating at a fixed prediction, the extrapolation points (marked in red) now follow a distinguishable trend that aligns better with the experimental values. This highlights a core advantage of dimensionless modelling: the input space, derived from physical principles, better reflects the multiscale interactions governing the sonochemical activity. Consequently, even when confronted with new frequency or power settings beyond the training domain, the model can still infer plausible output trends rather than reverting to static predictions. The validation set 1, set 2, and set 3 were generated under experimental setups that shared the same reactor geometry (i.e. cylindrical reactors with an inner diameter of 6.7 cm) as the training data. This geometrical consistency ensures similar acoustic field distributions and cavitation dynamics, allowing the model to generalize well across these datasets [7]. Predictions for these sets align closely with the y = x ideal prediction line, indicating strong generalization within structurally similar experimental domains (Fig. 10 (a − c)).

The validation Set 4 employed another reactor configuration with a decreased inner diameter of 5.0 cm [40]. Although the inputs are dimensionless, the modified vessel geometry changes in acoustic field distributions, resonance characteristics, and cavitation efficiency, which are not explicitly considered in the current model. This results in noticeable prediction discrepancies (yellow squares in Fig. 10 (d)), indicating that model efficacy is sensitive to geometry when extrapolating beyond the structural scale described in the training data. Notably, the model successfully predicted the trend of ultrasonic oxidation with changing initial temperatures, even though temperature was not explicitly included in the training dataset (red square in Fig. 10 (d)). This capability arises from dimensionless variables that implicitly encode thermal effects through physical parameters such as vapour pressure, solution properties and sound velocity, which are inherently temperature-dependent [17]. In contrast, models developed under the dimensional strategy failed to capture this effect due to the absence of temperature variation in the training space.

The combined dimensionless-machine modelling strategy displays enhanced generalisability and robustness across various sonochemical tasks and parameter combinations. By incorporating fundamental physical mechanisms through dimensionless variables and leveraging the nonlinear learning capacity of ML, the framework reduces dependence on individual scales, facilitates more stable training, and improves predictive applicability. Current limitations emerge when extrapolating to systems with different reactor geometries, as evidenced by the lower accuracy in validation Set 4. It exposes the model's sensitivity to reactor geometries not explicitly addressed in the current feature space. Further studies are encouraged to incorporate geometric descriptors and expand the training dataset to include various reactor configurations. This process will help overcome current limitations and enable more robust and scalable predictive modelling in sonochemistry.

### 3.5 Exploring model-learned mechanisms in sonochemistry using SHAP analysis

**Fig. 11:**
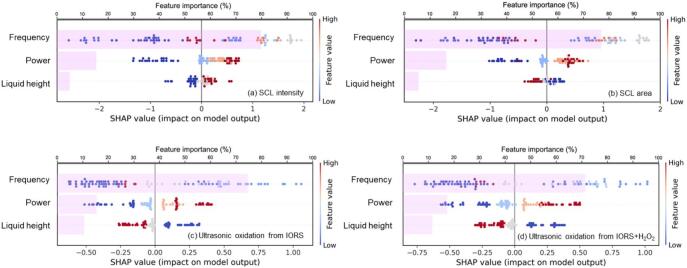
SHAP-based interpretation of dimensional-input models for sonochemical activities: SCL intensity (a), SCL area (b), ultrasonic oxidation from IORS (c), and ultrasonic oxidation from IORS + H_2_O_2_ (d).

**Fig. 12:**
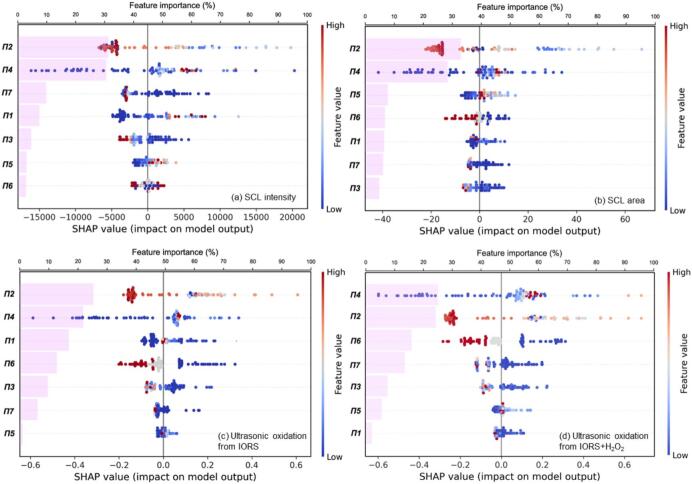
SHAP-based interpretation of dimensionless-input models for sonochemical activities: SCL intensity (a), SCL area (b), ultrasonic oxidation from IORS (c), and ultrasonic oxidation from IORS + H_2_O_2_ (d).

CatBoost was selected as the baseline model due to its strong predictive performance and superior interpretability through SHAP analysis [54]. By combining SHAP (for local interpretability) and feature importance (for global contribution), the model’s decision-making process can be effectively explained at both the individual and overall levels [55]. This type of feature attribution is not accessible to regression-based approaches, which are constrained by predefined functional forms and often require piecewise fitting to approximate nonlinear behaviours. Therefore, the CatBoost model provides predictive accuracy and mechanistic insights, bridging the gap between data-driven modelling and physical interpretation. Fig. 11, Fig. 12 show the importance and shapely value contribution of individual input variables, dimensional (Fig. 11) and dimensionless (Fig. 12), on the four objective outputs. The bars represent the importance of each feature across the full training set, (top axis) and the SHAP scatter points reveal the impact of feature values on individual predictions (bottom horizontal axis).

#### 3.5.1 Model-learned mechanisms under dimensional inputs

Ultrasonic frequency is the predominant variable in all dimensional-input models, contributing 79.5 % in SCL intensity, 77.9 % in SCL area, 73.9 % in ultrasonic oxidation from IORS, and 71.1 % in ultrasonic oxidation from IORS + H_2_O_2_. While frequency contributes over 70 % to total feature importance in all models, its SHAP distribution shows a nonlinear pattern (Fig. 11): positive in the mid-frequency range (light-coloured scatters) but negative at both low (deep blue) and high (deep red) frequencies. Moderate frequencies optimise the balance of cavitation bubble quantity, dimensions, and collapse energy conditions, conducive to effective sonochemical activation. This insight aligns with sonochemical theory: low frequencies allow larger bubbles but fewer cavitation events, whereas high frequencies limit bubble size and collapse intensity, reducing energy concentration [6,7]. The SHAP-based findings, therefore, offer an interpretable and mechanistically sound explanation for why mid-frequency regimes exhibit superior performance in SCL intensity and ultrasonic oxidation. The relatively low Pearson correlation coefficients between frequency and objective outputs (Fig. 6) emphasise the nonlinear characteristics of this connection. The regular linear analysis fails to recognise the importance of frequency since it fails to account for the nonlinear effects.

Ultrasonic power, the direct energy input controlling the sound pressure amplitude and supporting cavitation, effectively influences sonochemical activity. SHAP analysis indicates that model predictions (e.g., SCL intensity in Fig. 11 (a)) increase with power input in the low-to-moderate range. However, this relationship is not strictly linear. At higher power levels (dark red), SHAP values tend to plateau and resemble those observed at moderate power (Fig. 11), suggesting diminishing marginal returns. This pattern aligns with the theoretical “power threshold effect” in sonochemistry [7], wherein excessive power density (the ratio of power to volume) ceases to boost cavitation and could limit activity due to factors such as bubble coalescence, acoustic decoupling, and cavitation shielding [38,63]. Furthermore, Fig. 11 suggests that liquid height (volume) modulates the impact of ultrasonic power. For instance, under low liquid height conditions, the model captures signs of decreasing returns as in partially high power conditions, possibly a manifestation of threshold effects, where excessive power density leads to cavitation saturation or shielding. These SHAP patterns indicate that the CatBoost model can implicitly learn such trade-offs, reflecting an internalised understanding of energy density as a key regulator of cavitation.

CatBoost model successfully captures nonlinear dynamics which influence sonochemical systems. The represented feature-response contributions, including frequency-dependent cavitation and the power-volume trade-off, are in line with accepted sonochemical theories. This confirms the predictive and explanatory capabilities of the CatBoost, establishing it as a robust and insightful instrument for modelling complex physical processes in sonochemistry.

#### 3.5.2 Model-learned mechanisms under dimensionless inputs

Among all dimensionless variables, *Π2* and *Π4* consistently ranked as the most important features across all four prediction tasks (Fig. 12). In the SCL intensity model, *Π2* and *Π4* represented 31.32 % and 30.62 % of the overall feature importance, respectively. In the SCL area, their contributions were 32.49 % (*Π2*) and 28.00 % (*Π4*). In the model modelling ultrasonic oxidation from IORS, *Π2* accounted for 26.61 % and *Π4* for 22.85 %. In the IORS + H_2_O_2_ oxidation model, *Π4* and *Π2* remained dominant with 26.27 % and 25.13 %, respectively. *Π2*, defined as the ratio of water vapour molecules to total gas molecules inside the cavitation bubble (*n_w_*/*n_t_*), characterizes the bubble's internal thermal composition and reactivity potential. *Π4*, representing the applied ultrasonic power normalized by frequency and surface tension (*I_A_*/*fσ*), reflects the amount of acoustic energy available to drive cavitation.

Further examining the model's learned representation of *Π2* and *Π4*, the SHAP distributions reveal distinct nonlinear trends that align well with established sonochemical theories (Fig. 12). *Π2′s* SHAP distribution shows a clear peak pattern: SHAP values are highest at intermediate levels of *Π2 (*light coloured scatters), while both low and high values are associated with reduced or even negative contributions (deep-coloured scatters). This pattern reflects the thermal buffering effect of water vapour [64]. At moderate levels, water vapour supports cavitation reactivity by facilitating radical formation; however, excess vapour can absorb the thermal energy released during bubble collapse, suppressing local temperature peaks and thus reducing reactive oxygen species (including IORS and H_2_O_2_) generation. Conversely, too little vapour limits the availability of radical precursors [65]. The model has internalized this trade-off, capturing the optimal range of *Π2* for effective sonochemical activation.

Similarly, *Π4* exhibits a nonlinear SHAP distribution. In all models, low to moderate values of *Π4* are associated with increasingly positive SHAP contributions, confirming that enhanced ultrasonic power promotes sonochemical activity. However, at high *Π4* values (deep red scatter), the SHAP values plateau or slightly decline, suggesting diminishing marginal returns. This is consistent with the “power threshold” phenomenon in sonochemistry [7,63]. The model's capability to represent these effects supports its effectiveness in capturing complex nonlinear dynamics in sonochemistry.

In addition to *Π2* and *Π4*, several other dimensionless terms also exhibited non-negligible importance across different sonochemical tasks. These variables offer insights into secondary mechanisms and underscore key physical modulating factors in sonochemical systems. In the SCL-related models (Fig. 12 (a) and (b)), *Π2* and *Π4* dominate the global importance, contributing over 60 % of the feature importance. In contrast, for ultrasonic oxidation tasks (Fig. 12 (c) and (d)), although *Π2* and *Π4* remained important, the influence of other variables such as *Π6* (*P*_∞_*H*_R_/*σ,* ambient pressure), *Π3* (*c*/*H*_R_*f*, ultrasound propagation), and *Π7* (*C*_p_*T*_0_/(*H*_R_*f*)^2^, thermal dissipation) became more pronounced. This transition likely reflects the mechanistic complexity of chemical oxidation reactions, which are more sensitive to system pressure, thermal dynamics, and mass transport compared to instantaneous luminescent events [24,37]. Such differences highlight the distinct physical pathways governing each sonochemical output. Even within the same dimensionless framework, the importance of physical elements depends on the task, emphasising the necessity for response-specific analysis.

The CatBoost model demonstrates strong adaptability and interpretability across various sonochemical prediction tasks. SHAP-based interpretability results confirm that the decision patterns learned by the model align closely with established theoretical principles in sonochemistry, such as the thermal buffering and power threshold effects. This validates the applicability of the CatBoost model in sonochemical modelling and provide a means to visualize and corroborate hypothesized mechanisms, thus establishing a mutually reinforcing link between theoretical understanding and data-driven modelling.

## 4 Conclusions

This study developed a machine learning framework that integrates physically derived dimensionless parameters into the CatBoost algorithm for modelling complex sonochemical systems. Four representative outputs were investigated: SCL intensity, SCL area, ultrasonic oxidation from IORS, and ultrasonic oxidation from IORS + H_2_O_2_.

First, compared to our previous regression-based study, which primarily demonstrated the conceptual validity of the *Π* terms, the present framework enables quantitative prediction across the full parameter space. In the regression models, piecewise fitting was required to approximate nonlinear behaviours, and the results could only reproduce overall trends rather than deliver accurate numerical predictions. By contrast, the CatBoost models achieved consistently higher performance than regression models, achieving R2 values of 0.87–––0.95 on reserved test sets, whereas regression models reached only 0.67–––0.90 on the full dataset and further required dataset-specific corrections to predict independent validation sets.

Second, when comparing the two machine learning input strategies, both dimensional and dimensionless models achieved comparable predictive accuracy on the test sets (R2 = 0.86–––0.95 for dimensional vs. 0.87–0.95 for dimensionless). However, the dimensionless framework showed clear advantages in robustness and generalisability. In particular, dimensionless-input models provided more reliable extrapolation across external datasets, alleviated plateau effects observed in the dimensional models, and revealed consistent hyperparameter configurations across different tasks. These results indicate that the dimensionless formulation yields a more uniform and physically consistent feature space, thereby enhancing model transferability and reducing dependence on dataset-specific scaling.

Beyond predictive gains, the dimensionless machine learning strategy provides interpretability that regression could not offer. SHAP analysis revealed nonlinear mechanistic patterns, such as the thermal buffering and power threshold effects, with the dominant variables associated with thermal buffering and energy input scaling jointly contributing over 50 % of feature importance across tasks. These insights transparently link model predictions with established sonochemical theories, offering explanatory power not accessible to regression coefficients.

In summary, the proposed machine learning framework based on dimensionless variables combines the strengths of physics-guided feature engineering with nonlinear modelling. It delivers accurate, interpretable, and generalisable predictions of sonochemical activity, offering both a practical tool for system optimisation and a data-driven means of uncovering mechanistic insights. Building on these strengths, future work may extend this framework to specific sonochemical reactions, where dimensionless performance metrics could be employed to optimise yield, selectivity, and energy efficiency under diverse operating conditions.

## CRediT authorship contribution statement

**Yucheng Zhu:** Writing – review & editing, Writing – original draft, Methodology, Investigation, Formal analysis, Data curation, Conceptualization. **Ruosi Zhang:** Methodology, Investigation. **Xueliang Zhu:** Visualization, Methodology, Investigation, Data curation. **Xuhai Pan:** Writing – review & editing, Supervision. **Michael Short:** Writing – review & editing. **Lian X. Liu:** Writing – review & editing, Supervision, Resources. **Madeleine J. Bussemaker:** Writing – review & editing, Supervision, Resources, Project administration, Methodology, Funding acquisition, Conceptualization.

## Declaration of competing interest

The authors declare that they have no known competing financial interests or personal relationships that could have appeared to influence the work reported in this paper.
