# Supplementary material for: Machine learning modelling of sonochemical systems using physically-derived dimensionless groups
Source: Ultrason Sonochem. 2025 Sep 30;122:107593. doi: 10.1016/j.ultsonch.2025.107593 (PMC12538411; doi:10.1016/j.ultsonch.2025.107593)
Supplement: Supplementary Data 1 [file mmc1.docx]

**Supplementary Information (SI)**

**Machine learning modelling of sonochemical systems using physically-derived dimensionless groups**

Yucheng Zhu^a,b^, Ruosi Zhang^a^, Xueliang Zhu^a^, Xuhai Pan^b,*^, Michael Short^a^, Lian X. Liu^a^, Madeleine J. Bussemaker^a,*^

^a^ School of Chemistry and Chemical Engineering, University of Surrey, Guildford, United Kingdom

^b^ College of Safety Science and Engineering, Nanjing Tech University, Nanjing, China

* Corresponding authors.

*E-mail address:* [xuhaipan@njtech.edu.cn](mailto:xuhaipan@njtech.edu.cn) (Xuhai. Pan), [m.bussemaker@surrey.ac.uk](mailto:m.bussemaker@surrey.ac.uk) (Madeleine J. Bussemaker)

**Appendix A** Clarification of *Π* terms

**Table S1.** Definitions, symbols, and SI units of the parameters used in the dimensionless Π-term expressions

| Symbol | Description | SI Unit |
| --- | --- | --- |
| *ρ_L_* | Density of liquid | kg·m^-3^ |
| *n_t_* | Number of total molecules inside the bubble | mol |
| *n_w_* | Number of vapour molecules inside the bubble | mol |
| *c_L_* | Sound speed in the liquid | m·s^-1^ |
| *T_0_* | Initial temperature of liquid | K |
| *σ* | Surface tension of liquid | kg·s^-2^ |
| *μ_L_* | Dynamic viscosity of liquid | kg·m^-1^·s^-1^ |
| *P_∞_* | Static ambient pressure | kg·m^-1^·s^-2^ |
| *I_A_* | Acoustic intensity | kg·s^-3^ |
| *f* | Frequency of the exciting irradiation | s^-1^ |
| *H_R_* | Liquid height within the reactor | m |
| *C_p_* | Specific heat capacity of liquid | m^2^·s^-2^·K^-1^ |

**Table S2.** Definitions and physical significance of the dimensionless variables (*Π1*–*Π7*)

| *Π* term | Expression | Category | Physical meaning |
| --- | --- | --- | --- |
| *Π1* | *ρ*_L_*H*_R_^3^*f*^2^/*σ* | Bubble dynamics | Inertial-to-surface tension ratio |
| *Π2* | *n*_w_/*n*_t_ | Cavitation environment | Vapour-to-total gas ratio inside bubble |
| *Π3* | *c*/*H*_R_*f* | Acoustic wave transfer | Acoustic wavelength relative to reactor size |
| *Π4* | *I*_A_/*fσ* | Bubble dynamics | Acoustic intensity relative to surface energy |
| *Π5* | *μ*_L_*H*_R_*f*/*σ* | Bubble dynamics | Viscous damping contribution |
| *Π6* | *P*_∞_*H*_R_/*σ* | Cavitation environment | Ambient pressure influence |
| *Π7* | *C*_p_*T*_0_/(*H*_R_*f*)^2^ | Thermal effect | Thermal capacity and energy buffering |

**Appendix B** Selection of machine learning algorithms

KNN is a non-parametric, instance-based learning algorithm that predicts outcomes by averaging the outputs of the k most similar data points in feature space [1]. Its simplicity and ability to exploit local similarities make it suitable for small experimental datasets.

Linear regression assumes a direct linear relationship between the input features and the target output [2]. This model is simple, interpretable, and computationally efficient. While sonochemical systems are inherently complex and nonlinear [3], data preprocessing may reveal approximately linear patterns in specific cases. For this reason, linear regression was retained in the initial model screening as a simple yet informative candidate.

SVR uses kernel functions to map input data into higher-dimensional spaces, enabling flexible modelling of nonlinear relationships [4]. It is particularly well-suited for small to medium-sized datasets with complex parameter interactions. Given the limited size and experimental origin of the dataset used in this study, SVR is theoretically well-aligned with the characteristics of sonochemical modelling and offers strong potential for capturing nonlinearity in cavitation-driven systems.

Random forest is an ensemble learning algorithm that bases the bagging strategy (bootstrap aggregating) on training several decision trees on randomly resampled subsets of the data. The final prediction is made by averaging the outputs of all trees, which reduces overfitting and enhances generalization [5]. By integrating diverse decision paths, the random forest offers strong robustness and is particularly suitable for modelling multifactor responses and parameter interactions, which are commonly known in sonochemical systems.

Gradient boosting builds decision trees sequentially, where each new tree is trained to correct the prediction errors made by the previous ones [6]. This iterative learning process enables the model to improve its accuracy gradually. It is particularly effective in capturing complex nonlinear patterns and high-order feature interactions.

XGBoost is a highly optimized version of gradient boosting with regularization, column sampling, and parallel processing [7]. Known for its computational efficiency and strong performance on structured data, XGBoost is expected to perform well on sonochemical experimental datasets belonging to this dataset category.

CatBoost is a gradient boosting algorithm that incorporates ordered boosting and improved regularisation to overcome limitations in traditional frameworks, such as target leakage and training instability [8]. While initially developed for mixed-type data, it also performs strongly on numerical, structured datasets. CatBoost, characterised by rapid convergence, low sensitivity to feature scaling, and strong robustness, is potentially ideal for sonochemical data investigation.

More complex models such as neural networks were not included due to the limited dataset size, as deep architectures typically require large-scale data to avoid overfitting and achieve stable generalisation [9]. A smaller set of algorithms, in turn, would not adequately represent the diversity of modelling paradigms (parametric, kernel-based, ensemble, etc.), so a representative but balanced set of seven was adopted for screening.

**Appendix C** Sonochemical activities data review

This work used SCL and KI dosimetry to assess the 4 distinct sonochemical activities' index (Fig. S1). Due to its foundation in the iodine oxidation reaction, KI dosimetry analysis quantitative data that are more stable and objective [10, 11]. Consequently, the experimental dataset could be preserved unmodified for future study without additional processing (Fig. S1 c and d). Consequently, the experimental dataset could be preserved unmodified for future study without additional processing (Fig. S1 c and d). The quantification of SCL relies on the digital processing of visual data, which is vulnerable to noise signals and requires high-resolution images [12]. When the experiment is at an extremely high or low frequency, the intensity of the light signal is often weak, resulting in a low signal-to-noise ratio. In this case, the presence of noise signals may affect the accuracy of the quantified data. Therefore, strict data inspection is required to ensure the credibility of the data.


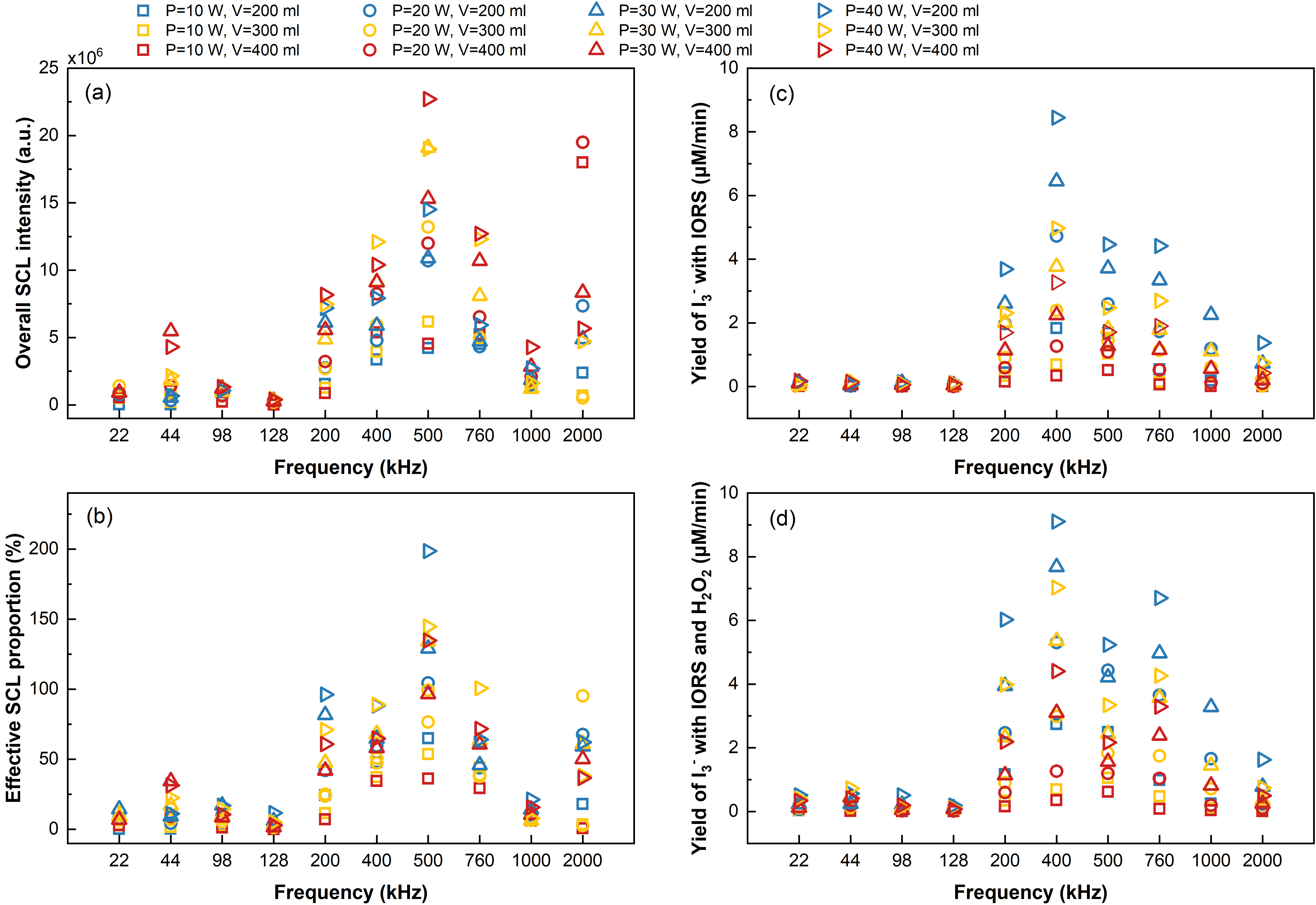


Fig. S1 Sonochemical activities measured by SCL (a and b) and KI dosimetry (c and d)

This work uses a relatively reliable SCL image quantification approach [13], yet particular attention is still required for some data points. The SCL data indicate anomalously elevated values at 2000 kHz (Fig. S1a and S1b), whereas the KI measuring method fails to detect similar high sound chemical activity under identical conditions. Despite the theoretical distinction between the two sonochemical processes, the occurrence of anomalous values caught our attention, prompting a more detailed examination of the experimental conditions associated with the elevated points in the SCL quantification data, which we then compared with the pertinent SCL luminescence images (Fig. S2). The analysis indicates that elevated SCL values at 44 kHz and 500 kHz correlate with the luminescence intensity observed in the experimental images (Fig. S2 a-d), suggesting that the quantification data can be preserved for model training. At 2000 kHz, despite the elevated SCL quantification value, the associated luminescence picture signal was poor and background noise was noticeable, suggesting that the high value may have resulted from noise interference rather than real SCL activity (Fig. S2 g-i). The utilisation of this data for SCL model training may distort the predictive outcomes.


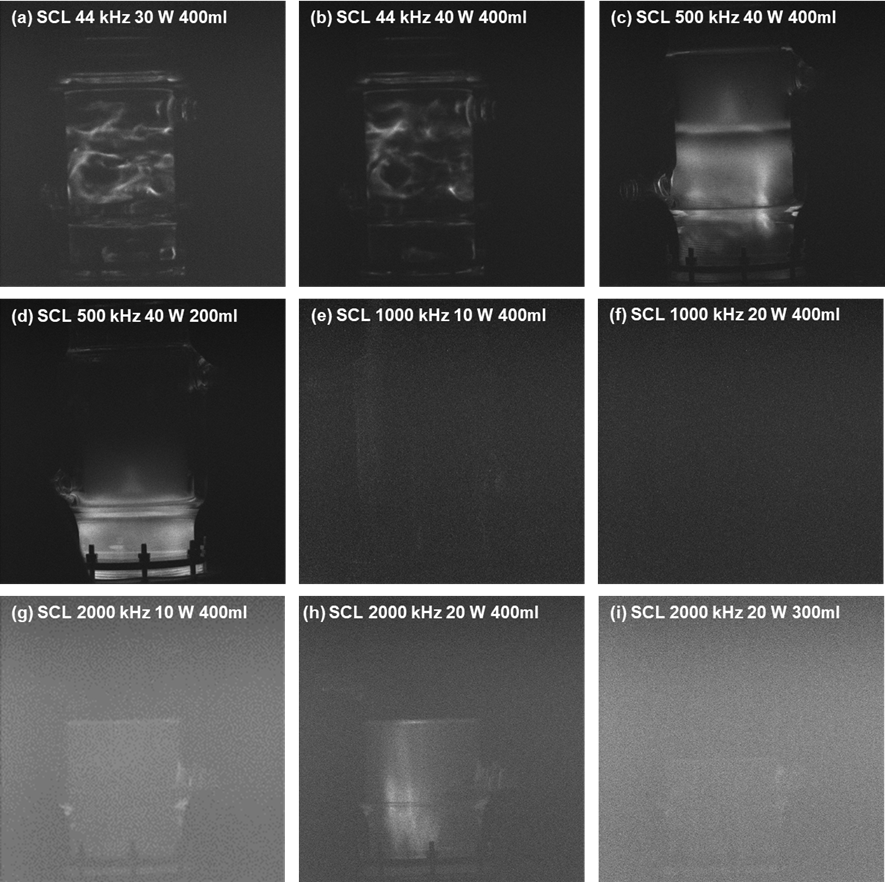


Fig. S2 SCL quantified data highlighted photo set

Furthermore, to enhance the validation of the SCL data under high-frequency settings, we conducted additional examinations of the images at 1000 kHz (Fig. S2 e and f). The findings indicate that the quantification data of the SCL under this operational situation is low, aligning with the observed situation. This suggests that the SCL data at 1000 kHz accurately represents the sonochemical activity and may thus be preserved in the model training dataset. Simultaneously, we assert that the frequency range of 22 kHz to 1000 kHz encompasses the majority of sonochemical reaction investigations. Consequently, to enhance the predictive accuracy of the SCL model, we choose to preserve solely the SCL data within the frequency range of 22 kHz to 1000 kHz for further modelling. Conversely, the test data of the KI dosimetry method is free from this limitation, allowing for the retention of the complete experimental data (22 kHz to 2000 kHz) for modelling.

**Appendix D** Baseline (machine learning) model selection

As shown in Table S3 and Fig. S3, all candidate models were first evaluated using five-fold cross-validation on datasets constructed from the dimensional input variables (frequency, power, and liquid height). The predictive performance was assessed through R², MAE, and MAPE. Among the seven models, ensemble learning methods, including random forest (bagging) and three boosting models (gradient boosting, XGBoost, CatBoost), demonstrate greater performance relative to non-ensemble models. The boosting approaches exhibited more consistency with low error and elevated R² across most tasks. For example, XGBoost achieved an R² of 0.89 for SCL intensity, and all boosting models maintained R² values above 0.90 for the other three targets, demonstrating robust and stable predictive performance. In contrast, KNN and linear regression show poor performance, with unstable or consistently low R² values and higher error metrics. Linear regression proved particularly poor for predicting SCL-related targets, with R² values of -0.77 ± 1.61 for SCL intensity and -0.28 ± 0.39 for SCL area, indicating ineffectiveness or invalid fits. Similarly, the KNN model consistently exhibited low predictive accuracy in sonochemistry, with R² values of 0.15 ± 0.18 and 0.21 ± 0.21 for predicting I₃⁻ yield with IORS and with IORS + H_2_O_2_, respectively. Although the input features were standardised by Z-score transformation and the target variables were transformed by the log1p function (see section 3.1.2), these preprocessing steps were insufficient to improve the performance of these models. Their inherent limitations in capturing nonlinear relationships and complex feature interactions make them less suitable for modelling sonochemical systems [3, 14].

**Table S3.** Performance comparison of candidate models for preliminary screening (with dimensional inputs)

| Target | Model | R^2^ | MAE | MAPE |
| --- | --- | --- | --- | --- |
| SCL intensity | KNN | 0.55±0.08 | 8224±1790 | 1.18±0.39 |
|  | Linear regression | -0.77±1.61 | 14283±4918 | 1.97±0.83 |
|  | SVR | 0.88±0.05 | 3660±667 | 0.40±0.10 |
|  | Random forest | 0.77±0.10 | 5751±1725 | 1.50±0.74 |
|  | Gradient boosting | 0.92±0.04 | 2866±542 | 0.29±0.06 |
|  | XGBoost | 0.89±0.06 | 3444±530 | 0.50±0.19 |
|  | CatBoost | 0.91±0.03 | 3303±798 | 0.42±0.18 |
| SCL area | KNN | 0.51±0.12 | 18.01±4.37 | 1.68±0.77 |
|  | Linear regression | -0.28±0.39 | 28.70±6.13 | 2.77±1.46 |
|  | SVR | 0.89±0.04 | 7.74±1.77 | 0.59±0.37 |
|  | Random forest | 0.67±0.15 | 13.91±3.93 | 1.95±1.30 |
|  | Gradient boosting | 0.92±0.04 | 6.18±1.28 | 0.37±0.12 |
|  | XGBoost | 0.90±0.04 | 6.68±2.12 | 0.71±0.73 |
|  | CatBoost | 0.91±0.04 | 6.67±2.83 | 0.48±0.26 |
| Yield of I_3_^-^ with IORS | KNN | 0.15±0.18 | 0.86±0.23 | 8.83±1.72 |
|  | Linear regression | 0.10±0.08 | 0.93±0.22 | 8.06±0.48 |
|  | SVR | 0.87±0.06 | 0.31±0.13 | 1.16±0.47 |
|  | Random forest | 0.50±0.19 | 0.56±0.23 | 3.50±0.79 |
|  | Gradient boosting | 0.95±0.02 | 0.20±0.06 | 1.03±0.42 |
|  | XGBoost | 0.92±0.05 | 0.24±0.11 | 1.01±0.30 |
|  | CatBoost | 0.93±0.07 | 0.20±0.12 | 1.08±0.47 |
| Yield of I_3_^-^ with IORS+H_2_O_2_ | KNN | 0.21±0.21 | 1.18±0.28 | 5.00±1.70 |
|  | Linear regression | 0.11±0.07 | 1.24±0.25 | 4.76±1.01 |
|  | SVR | 0.87±0.04 | 0.44±0.15 | 0.87±0.33 |
|  | Random forest | 0.55±0.14 | 0.75±0.26 | 2.38±0.58 |
|  | Gradient boosting | 0.93±0.04 | 0.31±0.06 | 1.21±0.58 |
|  | XGBoost | 0.92±0.04 | 0.31±0.11 | 0.69±0.18 |
|  | CatBoost | 0.94±0.04 | 0.28±0.12 | 0.77±0.34 |





**Fig. S3** Performance of candidate models with 3 dimensional inputs: SCL intensity (a-c), SCL area (d-f), Yield of I_3_^-^ with IORS (g-i) and Yield of I_3_^-^ with IORS+H_2_O_2_ (j-l).

The modelling strategy using dimensionless input variables reconfirmed the benefits of ensemble learning methods for predictive tasks in sonochemical systems, as demonstrated by the performance of candidate models (Table S4 and Fig. S4). CatBoost, Gradient boosting and XGBoost consistently displayed superior predictive performance, as indicated by higher R² and lower error metrics (MAE and MAPE) across distinct sonochemical outputs. For example, CatBoost achieved an R² of 0.91 for SCL area and 0.91 for the prediction of I_3_^-^ yield with IORS+H_2_O_2_, with corresponding MAPE values as low as 1.07 and 1.38, respectively. These results highlight its ability to capture the intricate nonlinear relationships in sonochemical systems accurately. Conversely, KNN and linear regression consistently demonstrated lower performances, For instance, linear regression resulted in an R^2^ of only 0.27 for I_3_^-^ yield with IORS and a relatively high MAPE of 13.48, compared to 0.91–1.66 for ensemble models. Meanwhile, KNN resulted in an R^2^ of just 0.39 and an MAE exceeding 9000 for SCL intensity, highlighting its inability to model the sonochemical system effectively. These results exposing their weaknesses in modelling complex nonlinear connections in acoustic systems.

**Table S4.** Performance comparison of candidate models for preliminary screening (with dimensionless inputs)

| Target | Model | R^2^ | MAE | MAPE |
| --- | --- | --- | --- | --- |
| SCL intensity | KNN | 0.39±0.16 | 9062±1895 | 1.77±0.99 |
|  | Linear regression | 0.07±0.18 | 11450±2986 | 1.96±0.76 |
|  | SVR | 0.85±0.05 | 4085±707 | 0.54±0.24 |
|  | Random forest | 0.58±0.12 | 7148±2272 | 1.52±0.74 |
|  | Gradient boosting | 0.88±0.03 | 3602±998 | 0.43±0.19 |
|  | XGBoost | 0.89±0.04 | 3560±994 | 0.48±0.20 |
|  | CatBoost | 0.89±0.04 | 3341±828 | 0.51±0.25 |
| SCL area | KNN | 0.39±0.20 | 20.20±4.11 | 4.41±2.97 |
|  | Linear regression | 0.21±0.10 | 23.68±5.10 | 6.05±4.49 |
|  | SVR | 0.85±0.04 | 8.79±2.06 | 0.99±0.56 |
|  | Random forest | 0.63±0.10 | 15.52±3.41 | 2.72±1.35 |
|  | Gradient boosting | 0.90±0.02 | 7.33±1.56 | 1.29±1.36 |
|  | XGBoost | 0.90±0.06 | 6.98±2.19 | 0.66±0.29 |
|  | CatBoost | 0.91±0.04 | 7.42±2.47 | 1.07±0.62 |
| Yield of I_3_^-^ with IORS | KNN | 0.44±0.21 | 0.16±0.05 | 5.76±2.84 |
|  | Linear regression | 0.27±0.18 | 0.22±0.05 | 13.48±4.92 |
|  | SVR | 0.86±0.06 | 0.08±0.04 | 1.26±0.47 |
|  | Random forest | 0.39±0.24 | 0.18±0.05 | 7.13±2.15 |
|  | Gradient boosting | 0.90±0.03 | 0.07±0.02 | 1.66±0.62 |
|  | XGBoost | 0.88±0.06 | 0.08±0.03 | 0.91±0.38 |
|  | CatBoost | 0.91±0.03 | 0.07±0.02 | 1.65±0.77 |
| Yield of I_3_^-^ with IORS+H_2_O_2_ | KNN | 0.34±0.35 | 0.23±0.06 | 4.60±2.02 |
|  | Linear regression | 0.27±0.17 | 0.29±0.06 | 9.49±4.06 |
|  | SVR | 0.83±0.06 | 0.13±0.04 | 1.04±0.45 |
|  | Random forest | 0.40±0.27 | 0.24±0.06 | 4.50±1.64 |
|  | Gradient boosting | 0.90±0.04 | 0.09±0.02 | 1.33±0.89 |
|  | XGBoost | 0.84±0.04 | 0.12±0.02 | 0.66±0.26 |
|  | CatBoost | 0.90±0.05 | 0.10±0.03 | 1.38±0.53 |





**Fig. S4** Performance of candidate models with 7 dimensionless inputs: SCL intensity (a-d), SCL area (e-h), Absorbance in KI solution (i-l) and Absorbance in catalyst-assisted KI solution (m-p)

**Supplementary references**

[1] R.K. Halder, M.N. Uddin, M.A. Uddin, S. Aryal, A. Khraisat, Enhancing K-nearest neighbor algorithm: a comprehensive review and performance analysis of modifications, Journal of Big Data, 11 (2024) 113. <https://doi.org/10.1186/s40537-024-00973-y>

[2] N.R. Draper, H. Smith, Applied regression analysis, John Wiley & Sons, 1998.

[3] X. Wang, Z. Ning, M. Lv, C. Sun, Machine learning for predicting the bubble-collapse strength as affected by physical conditions, Results in Physics, 25 (2021) 104226. <https://doi.org/10.1016/j.rinp.2021.104226>

[4] A.J. Smola, B. Schölkopf, A tutorial on support vector regression, Statistics and computing, 14 (2004) 199-222. <https://doi.org/10.1023/B:STCO.0000035301.49549.88>

[5] L. Breiman, Random forests, Machine learning, 45 (2001) 5-32. <https://link.springer.com/article/10.1023/a:1010933404324>

[6] J.H. Friedman, Greedy function approximation: a gradient boosting machine, Annals of statistics, (2001) 1189-1232. <https://www.jstor.org/stable/2699986?seq=1>

[7] Xgboost: A scalable tree boosting system,·.·<https://doi.org/10.1145/2939672.2939785>

[8] L. Prokhorenkova, G. Gusev, A. Vorobev, A.V. Dorogush, A. Gulin,·CatBoost: unbiased boosting with categorical features,·in:·Advances in neural information processing systems,·2018,·pp.·<https://proceedings.neurips.cc/paper_files/paper/2018/file/14491b756b3a51daac41c24863285549-Paper.pdf>

[9] Neural networks and machine learning,·IEEE,·

[10] S. Merouani, O. Hamdaoui, F. Saoudi, M. Chiha, Influence of experimental parameters on sonochemistry dosimetries: KI oxidation, Fricke reaction and H2O2 production, Journal of Hazardous Materials, 178 (2010) 1007-1014. <https://doi.org/10.1016/j.jhazmat.2010.02.039>

[11] R.J. Wood, J. Lee, M.J. Bussemaker, Disparities between sonoluminescence, sonochemiluminescence and dosimetry with frequency variation under flow, Ultrasonics Sonochemistry, 58 (2019) 104645. <https://doi.org/10.1016/j.ultsonch.2019.104645>

[12] T.J. Tiong, T. Chandesa, Y.H. Yap, Comparison of sonochemiluminescence images using image analysis techniques and identification of acoustic pressure fields via simulation, Ultrasonics sonochemistry, 36 (2017) 78-87. <https://doi.org/10.1016/j.ultsonch.2016.11.003>

[13] Y. Zhu, X. Zhu, X.-H. Pan, L. Liu, M. Bussemaker, Correlation of sonochemical activities measured via dosimetry and an area-selective analysis of sono (chemi) luminescence, RSC Mechanochemistry, (2025). <https://pubs.rsc.org/en/content/articlehtml/2025/mr/d5mr00006h>

[14] M. Mailagaha Kumbure, P. Luukka, A generalized fuzzy k-nearest neighbor regression model based on Minkowski distance, Granular Computing, 7 (2022) 657-671. <https://doi.org/10.1007/s41066-021-00288-w>
